# Hepatectomy guided by indocyanine green fluorescent imaging for visualizing bile leakage (with video)

**DOI:** 10.1002/ccr3.5942

**Published:** 2022-06-03

**Authors:** Takehiko Hanaki, Naruo Tokuyasu, Teruhisa Sakamoto, Yoshiyuki Fujiwara

**Affiliations:** ^1^ Department of Gastrointestinal and Pediatric Surgery School of Medicine Tottori University Faculty of Medicine Yonago Japan

**Keywords:** biliary fistula, fluorescence imaging, hepatectomy, indocyanine green, navigation surgery

## Abstract

Using indocyanine green (ICG), a standard reagent used in liver function tests, bile leaks from exfoliated liver sections can be detected with higher sensitivity than naked‐eye observation. This presentation will introduce the technique of using ICG to detect bile leaks that cannot be detected by the naked eye.

## Graphical abstract

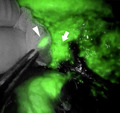

## 1 INTRODUCTION

Biliary fistulas (BFs) after hepatectomy are a challenging complication and are occasionally accompanied by serious conditions, such as liver failure and severe infection, despite recent improvements in perioperative care and surgical techniques. The bile leak test has been reported to be efficient in detecting bile leakage during surgery^1^; however, it is not valid for identifying bile leaks that do not involve the common bile duct (e.g., Nagano Type D bile leakage^2^).

**FIGURE 1:**
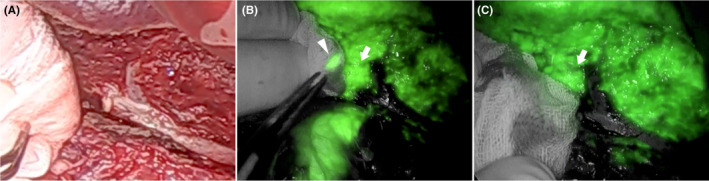
Observation of the liver section plane. On naked‐eye observation, the gauze that was pressed against the section plane of the liver was not stained yellow with bile (A). Near‐infrared observation showed an indocyanine green fluorescent spot (arrowhead) from the parenchyma of the liver section (arrow) (B). A Z‐shaped suture was added to the site of contamination (arrow) in the liver section plane, and it can be seen that the fluorescent spot of gauze disappeared (C)

In our institution, indocyanine green (ICG) (10 mg/body) is administered intravenously to determine the hepatic resection area, and the liver is observed using an ICG camera system (Stryker AIM1588) after the completion of the hepatectomy. As intravenous ICG is taken up by the hepatocytes and is gradually excreted in bile, this property can be used to identify the bile ducts. In the section plane obtained after hepatectomy, bile leakage could be observed more clearly as an ICG fluorescence contamination of the gauze compared with naked‐eye observation (Figure 1, Video S1). This allows for the appropriate treatment of intraoperative subclinical bile leakage, thus preventing BFs. Further, the method seems beneficial for detecting Nagano Type D bile leakage,^2^ which cannot be detected using conventional bile leak tests.

## CONFLICT OF INTEREST

The authors have no conflicts of interest or financial ties to disclose.

## AUTHOR CONTRIBUTIONS

TH: collected the patient data, performed surgery and a literature review, and wrote the manuscript; NT and TS: revised the manuscript; YF: was involved in overall supervision of the study. All authors have read and approved the final version of the manuscript.

## CONSENT

Written consent for this presentation was obtained from the patients who appeared in the figure and supplementary video.

## Supporting information

Video S1
